# Structural Equation Modeling (SEM) and Temporal Dominance of Sensations (TDS) in the Evaluation of DOC Douro Red Wine’s Sensory Profile

**DOI:** 10.3390/foods11081168

**Published:** 2022-04-18

**Authors:** Elisete Correia, Eduardo Amorim, Alice Vilela

**Affiliations:** 1 Center for Computational and Stochastic Mathematics (CEMAT), Department of Mathematics, University of Trás-os-Montes and Alto Douro, Apt. 1013, 5001-801 Vila Real, Portugal; ecorreia@utad.pt; 2 University of Trás-os-Montes e Alto Douro, Apt. 1013, 5001-801 Vila Real, Portugal; edufamorim@hotmail.com; 3 CQ-VR, Chemistry Research Center, Department of Biology and Environment, School of Life Sciences and Environment, University of Trás-os-Montes and Alto Douro, Apt. 1013, 5001-801 Vila Real, Portugal

**Keywords:** sensory profile, single point techniques, temporal dominance methods, red wine, douro region

## Abstract

In the Portuguese Douro region, several DOC (Denomination of Controlled Origin) Douro red wines are produced and, due to the peculiar characteristics of the three Douro sub-regions, present particular imprinted terroirs, that can be perceived when tasted. Considering the DOC Douro wine’s sensory profile and terroir, this study aimed to analyze the sensory characteristics of red wines produced in the three Douro sub-regions (Baixo Corgo, Cima Corgo, and Douro Superior) by a single point sensory technique, a Quantitative Descriptive Analysis—QDA^®^ and also applying a temporal method-TDS (Temporal Dominance of Sensations). The use of QDA and TDS methods proved to be efficient in the wine’s sensory profile characterizing. The QDA^®^ method allowed a detailed classification of attributes; however, the TDS method proved to be much more efficient. Moreover, the wines of the three sub-regions presented profiles with characteristics very similar in olfactory and taste/flavor aspects, pointing out a huge relation between the characteristics of the three sub-regions and the grape varieties present in the wines. Globally, the olfactory profile of wines is characterized by Fruity, Floral, and Balsamic aromatic notes, while the taste/flavor profile stands out, highlighting Astringency and Acidity and, again, Fruity as the main in-mouth aroma. It was also possible to conclude that TDS is a fast method that is easy to apply and has excellent results in the evaluation of the olfactory and taste/flavor profile of wines and, with a larger set of samples, it would be possible to obtain characteristic TDS curves for each Douro sub-region, providing a wine’s fingerprint that could be used for authentication and traceability purposes.

## 1. Introduction

In wine production, quality and style are considered to be impacted by the place where the vineyards grow, and this relationship between the sensory and chemical attributes and the place of origin is called ‘terroir’ [1]. The imprinted “terroir” can be derived from a diverse range of factors related to the physical environment, such as climate, topography, soil, and geology, as well as human intervention, including viticulture and oenological decisions and practices [2].

**Figure 1:**
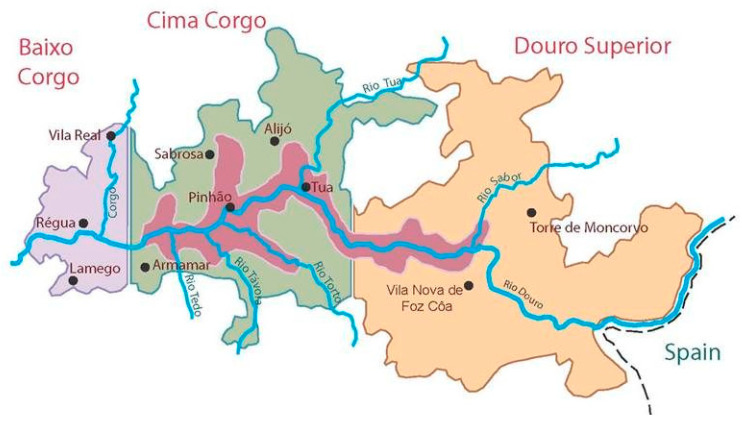
Sub-regions of the Demarcated Douro Region, Portugal.

The Douro region, Figure 1, in the north of Portugal, is the first demarcated and regulated wine region in the world since 1756. The region has a total area of about 250,000 hectares, of which 43,708 hectares with vineyards. It is divided into three sub-regions, Baixo Corgo, Cima Corgo, and Douro Superior. In 2001, the region received the World Heritage badge awarded by the United Nations Educational, Science and Culture Organization (UNESCO) [3].

The vinicultural zone covers the steep slopes along the banks of the lower reaches of the Douro River (Figure 1), which is one of the longest on the Iberian Peninsula. The Douro River finds its source in northern Spain, where it is called Duero. It flows through the vineyards of Ribera del Duero before finding the Portuguese border and becoming the Douro. The Douro’s most unifying peculiarity is its mountainous terrain. Typically, however, the vineyards stretch up the steep, dry slopes on either side of the river and its myriad tributaries on narrow rocky terraces.

The three sub-regions express different aspects of the area’s hot continental climate (Table 1). 

In the Douro region, in addition to the famous Port wine, several red table wines, also denominated DOC Douro red wines, are produced and, due to the peculiar characteristics of the three Douro sub-regions, present particular imprinted terroirs that can be perceived when tasted. The DOC Douro red wines tend to be robust and full-bodied and often spend some time aging in oak. The same red grape varieties that are present in Port wines are typically used for the dry wines, being the more important ones, the Touriga Nacional, Touriga Franca, and Tinta Roriz (also called Tempranillo in Spain), either in a blend or bottled alone. Young wines are often characterized by distinctive aromas: “caramel”, “honey”, “red-fruit” (e.g., red and black currant, cherry), “floral” (such as rose, violet), “nutty” (almond and hazelnut are the most cited), “balsamic”, and “resinous” [5]. Many of these descriptors appear on the wine labels and are mentioned in wine-related magazines such as the Portuguese Revista de Vinhos, or the international Wine Enthusiast and Wine Spectator magazines.

The characterization of the sensory profile of wines from a given region is a recurrent theme and has been addressed in several investigations [2,6,7]. Furthermore, descriptions of the sensory attributes of wine, usually generated by wine specialists, are widely used to guide consumer purchases [8]. Currently, descriptive analysis is based on the methods of Quantitative Descriptive Analysis (QDA^®^), used worldwide, and considered a reference for robust, reliable, and valid sensory analyses to measure sensory properties among a set of samples [9]. A recent study by Guld et al. [10] pointed to the QDA^®^ method as the most sensitive and detailed sensory analysis in wines compared to the OIV system of 100 points. However, the QDA^®^ process is time-consuming and expensive, as participants or panelists must be selected and trained, which can take several months [11].

Compared to traditional sensory descriptive analysis, the Temporal Dominance of Sensations (TDS) method can be seen as faster and less expensive, as it does not require any training step [12]. Relatively new in the sensory field, it consists of describing the temporal evolution of the different sensations developed during food and drink consumption [13]. Moreover, TDS is better than temporal dominance methods due to the possibility of consecutively recording several sensory attributes over time, identifying one specific attribute as “dominant” [14]. The method fills the gap between static multidimensional sensory profiles and time-intensity (TI) one-dimensional dynamics, offering a way to simultaneously evaluate multiple attributes, dynamically, over time [15].

Considering the DOC Douro wine’s sensory profile and terroir, this investigation aimed to analyze the sensory characteristics of red wines produced in the three Douro sub-regions (Baixo Corgo, Cima Corgo, and Douro Superior) by a single point sensory technique—QDA^®^- and also applying a temporal method-TDS, intending to verify if there are specific sensory profiles related to each of the sub-regions.

A Structural Equation Modeling (SEM) tool for the QDA^®^ data analysis was used. This statistical analysis is mainly used in ecology and forestry data analysis [16,17], psychology and other social sciences [18], and network meta-analysis [19]. Recently was used in the study of the sensory profile of Vinho Verde monovarietal wines [20]. This technique simultaneously combines confirmatory factor analysis (measurement model) and regression analysis (structural model), allowing (i) taking measurement error into account; (ii) incorporating unobserved variables with multiple indicators; (iii) modeling and testing complex patterns of relationships and, (iv) testing local and global assessment and specific assumptions about parameters [21].

## 2. Materials and Methods

### 2.1. Wines

Seeking a representative number of samples, eighteen wines from different producers were used, six distinct samples representing each of the three sub-regions of the DDR (Baixo Corgo, Cima Corgo, and Douro Superior), Table 2. The choice of the wines followed six criteria:

1. Have the DOC Douro (Designation of Origin Certification);
2. Be classified as red, dry, and still wine;
3. Contain mandatorily, but not exclusively, in the composition of the blend the grape varieties Touriga Franca, Touriga Nacional, and Tinta Roriz;
4. Be produced between 2015 and 2017;
5. Bottled in a 750 mL container and properly labeled and available for marketing;
6. With or without aging in oak barrels.

Most of the wines were directly awarded to the respective producers between April and December of 2019; others were purchased in specialty stores. All bottles were stored in an environment conducive to their conservation, in a horizontal position, at room temperature (20 ± 2 °C), and without light incidence. Table 1 shows the relationship and codification of wines together with some analytical data according to the technical data sheet provided by the producers.

### 2.2. Choice of Attributes

#### 2.2.1. For QDA^®^

The list of attributes selected for the development of the QDA^®^ tasting sheet (Supplemental Table S1) was adapted according to the works of Böhm [22], Almeida [23]; Stone and Sidel [24], and WSET Levels 3 and 4 Systematic Approach to Tasting Wine^®^¹ [25,26].

The attribute’s intensities were scored on a five-point scale (ranging from 1, lowest intensity, to 5, highest intensity), adapted from Monteiro et al. [27].

#### 2.2.2. For TDS

For the TSD, the list of olfactory attributes and gustatory attributes (Table 3) was obtained according to the highest citation frequency, by the tasters, in the sensory evaluations of QDA^®^.

### 2.3. Selection and Training of Tasters

Recruitment was undertaken through direct contact and referrals at the University of Trás-os-Montes and Alto Douro (UTAD). The selection of panels followed the procedures established according to the ABNT NBR ISO 8586 [28] standard. For the panel of trained tasters (PP1—trained panelists), twelve participants were selected between twenty candidates, seven women and five men, with ages between twenty-one and forty-seven years old. In the second panel (PP2—expert panelists), six tasters were recruited because they had previous experience in sensory panels. This panel was formed by three women, two sommelières with professional training and a master’s degree in enology and viticulture, and three men, all enologists, aged between twenty-five and forty-seven years old. All recruited panelists, trained and experts, answered the questionnaire for the selection, with the following criteria for approval:

1. Not having health problems or food allergies that did not allow them the consumption of alcoholic beverages;
2. Having participated in wine sensory panels previously;
3. Be a frequent consumer of red wine;
4. Having interest and availability to attend training sessions and tests.

The selected candidates participated in three training sessions lasting approximately two hours and thirty minutes each. The sessions were held in a tasting room with adequate conditions according to ISO 8589 [29]. Table 4 lists the content taught in the training sessions.

### 2.4. Tasting Procedure

#### 2.4.1. For QDA^®^

The sessions were held in the sensory analysis laboratory in the Enology Building at the University of Trás-os-Montes and Alto Douro (UTAD). The laboratory complies with all ISO 8589 [29] regulatory requirements, giving the panel an appropriate environment; such conditions are critical to ensure the quality and reliability of the results.

The tasters had at room temperature mineral water and dried unsalted biscuits to clean the palate and taste buds between the tasting of each wine, as well as spatters and napkins.

The wines were prepared in a support room, properly enveloped and coded with random three-digit codes so as not to identify the labels, and opened thirty minutes in advance of the tasting. Ten minutes before the start, without the presence of the tasters, fifty milliliters (50 mL) of each wine were served in ISO 3591 [30] tasting glasses, at room temperature (20 ± 2 °C), coded, and randomly arranged.

As soon as the tasters entered the test room, they were directed to their places, where they were instructed to sign the attendance list and fill out their data in the paper tasting forms, and then start the tasting.

Three sessions were performed for three days in a row. For PP1, in each session, six wines from the same sub-region were analyzed. For PP2, in each session, six wines were evaluated, two from each sub-region. The sessions of the panel of trained tasters (PP1) lasted approximately forty-five minutes, while those of PP2 lasted thirty minutes.

#### 2.4.2. For TDS

In TDS sessions, expert tasters (PP2 panel) used laptops with the free SensoMaker^®^ software (version 1.91, 2017, Universidade Federal de Lavras UFLA, Lavras, MG, Brazil) for data acquisition and analysis.

TDS evaluations were performed in three sessions, one per day. The sessions were composed of two types of analysis, olfactory and gustatory, evaluated sequentially. In each of the analyses, the tasters evaluated six wines, two from each subregion. The tasters followed the TDS assessment protocol (Table 5), and the sessions lasted approximately fifteen minutes.

### 2.5. Data Analysis

The homogeneity and performance of the trained panel (PP1) and the specialists (PP2) were evaluated by factor analysis (FA), on the correlation matrix, with the extraction of factors by the component method. The common factors retained were those that had an eigenvalue greater than one. To evaluate the validity of FA, the KMO criterion and Bartlett’s test of sphericity were used [21,32].

Structural equation modeling (SEM) was used to characterize the sensory profile of the wines of each of the three sub-regions of the DDR. For the estimation of the parameters, we used the maximum likelihood method, which provided the standardized estimates of the coefficients (an estimate higher than 0.5 in absolute value indicates a strong association). The existence of outliers was evaluated by the square distance of Mahalanobis (D^2^), and the normality of the variables was evaluated by the skewness (Sk) and kurtosis (Ku) coefficients. Comparative Fit Index (CFI), Goodness of Fit Index (GFI), Root-Mean-Square Error of Approximation (RMSEA), and χ^2^ statistics were used to determine the adequacy of the models. A CFI > 0.90, GFI > 0.90, and RMSEA < 0.05 with 90% CI < 0.10 are acceptable indices of fit for the model, and χ^2^/df < 2 is considered to be good [21].

Multivariate analysis of variance (MANOVA) was performed to evaluate the significance of the sub-regions on the quantitative parameters of TDS curves for olfactory and gustatory attributes. When MANOVA detected statistically significant effects, univariate analysis of variance (ANOVA) was performed, followed, whenever possible, by Tukey’s post-hoc test.

All statistical analyses were performed using SPSS 27.0 (IBM SPSS 27.0, Chicago, IL, USA) and AMOS (v. 22, SPSS, An IBM Company, Chicago, IL, USA) software.

## 3. Results

### 3.1. Panels (PP1 and PP2) Performance Assessment

The results obtained for the PP1 panel (Table 6) indicate good data adequacy [KMO = 0.9 and Bartlett’s test (*p* < 0.001)]. According to the rule of eigenvalue greater than 1 (λ = 10.766), the relational structure is explained by one factor, which explains 89.716% of the variability. Table 6 shows the factorial weights, communalities, eigenvalue, and percentage of variance explained for the factor.

As can be seen, all tasters present high loadings, which indicates similar behavior between them. Additionally, the communality value for each of the tasters is high, indicating that the component is adequate to describe the factorial structure among the tasters.

Regarding the PP2 panel (Table 7), as it was observed for the PP1, the FA is also adequate [KMO = 0.849, and Bartlett’s test (*p* < 0.001)]. The relational structure of the classifications is explained by one factor that presents an eigenvalue of 5.530 and explains 92.163% of the total variability.

The results obtained indicate a homogeneous behavior on the part of the panel of expert tasters (PP2). Moreover, the high value of the communalities is an indicator of the suitability of the component to describe the factorial structure among the expert tasters.

### 3.2. Sensory Profile of Wines Data Analysis by SEM

**Figure 2:**
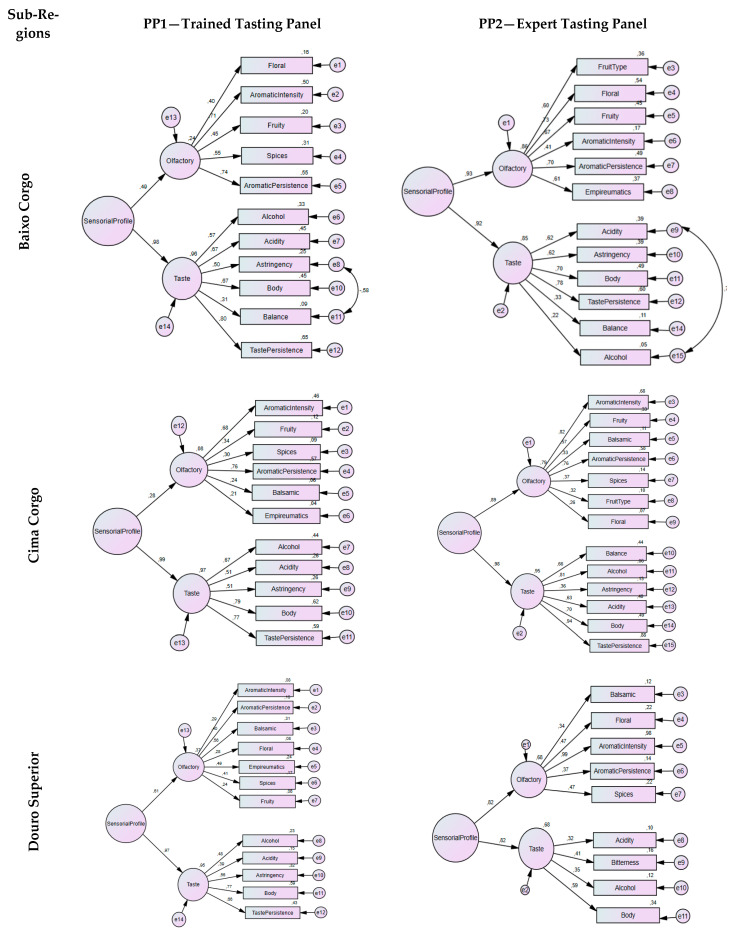
SEM schematic representation, standardized coefficients, and the individual reliability of each of the items in the final second-order model for the sensory profile of the wines of each of the three DDR sub-regions, for both tasting panels, PP1 and PP2.

After QDA analysis, a structural equations modeling methodology, SEM, was applied for the sensory profile characterization of the wines studied. Figure 2 shows the schematic representation and values of the standardized factor weights and the individual reliability of each of the items in the final second-order CFA model for the sensory profile of the wines. As can be seen, the measurement model of sensory attributes under study, for PP1 panel, revealed a good measurement fit for the sub-regions Baixo Corgo (χ^2^/df = 1.002; CFI = 1; GFI = 0.907; RMSEA = 0.005; P[rmsea < 0.05] = 0.744), Cima Corgo (χ^2^/df = 1.655; CFI = 0.814; GFI = 0.876; RMSEA = 0.092; P[rmsea < 0.05] = 0.045), and Douro Superior (χ^2^/df = 1.743; CFI = 0.681; GFI = 0.831; RMSEA = 0.102; P[rmsea < 0.05] = 0.012). Most descriptors have high factor weights and the taste/flavor sensation is the strongest in all models. Regarding the sensory profile of wines by expert tasters (PP2 panel), the results obtained for Baixo Corgo, Cima Corgo, and Douro Superior showed an acceptable measurement fit (χ^2^/df = 1.931; CFI = 0.741; GFI = 0.797; RMSEA = 0.163; P[rmsea < 0.05] = 0.003), (χ^2^/df = 1.647; CFI = 0.744; GFI = 0.0.706; RMSEA = 0.136; P[rmsea < 0.05] = 0.005), (χ^2^/df = 1.305; CFI = 0.825; GFI = 0.855; RMSEA = 0.093; P[rmsea < 0.05] = 0.263), respectively. We can also observe that most of the descriptors have high factorial weights and that both sensations manifest themselves equally in the sensory profile characterization.

In Figure 2, concerning the PP1 (trained tasting panel), we can also verify that, for Baixo Corgo, the olfactory attributes that contribute the most to the olfactory discrimination of wines are, *Aromatic Persistence*, *Aromatic Intensity*, *Spices*, *Fruity*, and *Floral*, ordered according to the standardized estimated values. Regarding the taste/flavor attributes: *Taste persistence*, *Acidity*, and *Body* with the same contribution, *Alcohol*, *Astringency*, and *Balance*, are those who contribute the most.

For the Cima Corgo sub-region, the olfactory attributes that contribute the most, ordered according to factor weights, are *Aromatic Persistence*, *Aromatic Intensity*, *Fruity*, *Spices*, *Balsamic*, and *Empireumatic*. In the taste/flavor attributes, we can find *Taste Persistence*, *Body*, *Alcohol*, *Astringency*, and *Acidity.*

Different characteristics present in the wines from the Douro Superior sub-region. In the olfactory group, we find *Balsamic*, *Empireumatic*, *Spices*, *Aromatic Persistence*, *Aromatic Intensity*, *Floral*, and *Fruity*, and among the taste/flavor attributes are *Body*, *Taste Persistence*, *Astringency*, *Alcohol*, and *Acidity* (Figure 2).

When the analysis was performed by a panel of experts (PP2), Figure 2, in the Baixo Corgo sub-region, the olfactory attributes, ordered according to factor weights, are *Aromatic Persistence*, *Floral*, *Fruity*, *Empireumatic*, *Aromatic Intensity*, and *Spices*, while, for taste/flavor, are *Taste Persistence*, *Body*, *Astringency*, and *Acidity*.

The wines from the Cima Corgo sub-region are characterized by the olfactory attributes of *Aromatic intensity*, *Aromatic persistence*, *Fruity*, *Spices*, *Balsamic*, *Floral*, and *Empireumatic* and by the taste/flavor attributes of *Taste Persistence*, *Alcohol*, *Body*, *Balance*, *Acidity*, and *Astringency*. In comparison, the sensory profile of Douro Superior sub-region wines is most characterized by *Aromatic intensity*, *Floral*, *Aromatic Persistence*, *Balsamic* (olfactory characteristics), *Body*, *Taste Persistence*, and *Balance* (taste/flavor attributes), Figure 2.

### 3.3. Sensory Profile of Wines Applying the TDS Evaluation

**Figure 3:**
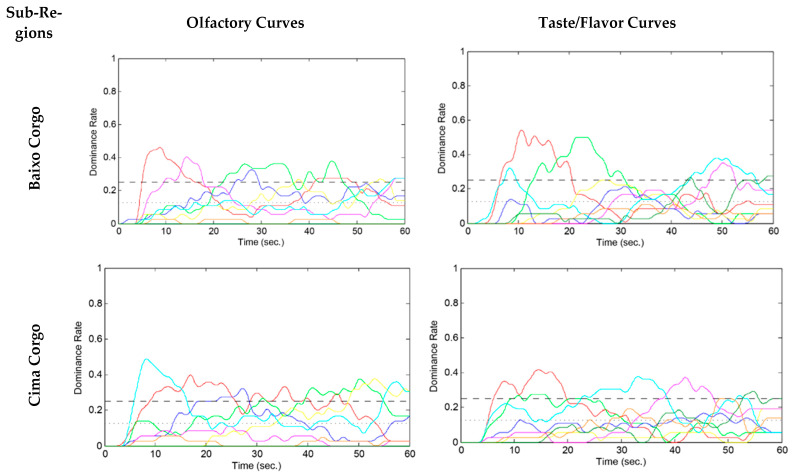

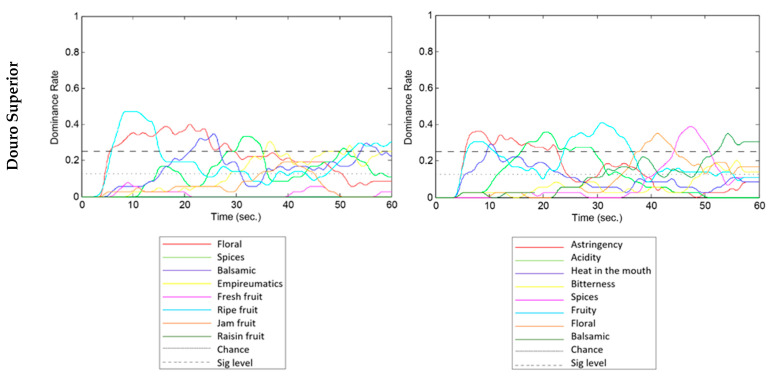
TDS curves of olfactory and gustatory attributes of the wines evaluated by the PP2 tasting panel. For each attribute, a colored line is presented in each graphic.

As it was mentioned before, TDS was performed using laptops with the free SensoMaker software (version 1.91, 2017) for data acquisition and analysis. The software output shows the Temporal Dominance of Sensation curves (Figure 3), along with some quantitative parameters of TDS curves such as DRmax (highest maximum dominance rate); T max (level line of significance), and T 90% max (maximum dominance rate), Table 8.

For each evaluated wine and each time, dominant rates were calculated by attributes [33]. These rates are obtained by dividing the number of citations of an attribute by the number of panelists and the number of replications. Since one panelist can have only a single dominant attribute at each time, the sum of the dominance rates over attributes is equal to one at each time; the higher the dominant index, the better the agreement among panelists. The graphics represent two other lines: (i) the “chance level” represents the dominance rate that an attribute can obtain by chance (1/number of attributes), and (ii) the “significance level line”, based on a binomial test, wish expresses the smallest value of the proportion being significantly higher than the chance level [33]. When the TDS curves go from between the chance and the significance levels to above the latter, they are consistent at the panel level [34].

In the olfactory evaluation of wines from the Baixo Corgo sub-region, according to the graphic representation of the TDS curves (Figure 3), six attributes overlapped with the significance level line, according to the perception of the tasters: *Floral*, *Fresh Fruit*, *Spices*, *Balsamic*, *Ripe Fruit*, and *Empireumatic*. The *Floral* attribute recorded the highest maximum dominance rate (DR max) with the representativeness of 46.09% of the evaluations, at 8.7 s (seconds) of the test, followed by attributes *Fresh fruit* (DR max 40.45%) at 14.3 s, *Spices* (DR max 37.88%) at 44.8 s, *Balsamic* (DR max 32.58%) at 28 s, *Ripe fruit* (DR max 27.78%) at 58.5 s and *Empireumatic* (DR max 27.02%) at 55s. The *Spices* attribute stood out with the largest range of the maximum dominance rate (T 90% max) lasting 20.1 s. As for the taste/flavor evaluation, the following attributes: *Astringency*, *Fruity*, *Acidity*, *Bitterness*, *Balsamic*, and *Spices*, overlapped the level line of significance and were ordered according to the perception of the tasters. The highest maximum dominance rate was recorded by the attribute *Astringency* (DR max 54.29%) at 10.6 s of the evaluation, followed by attributes: *Acidity* (DR max 50%) at 21.5 s, *Fruity* (DR max 38.13%) at 49 s, *Spices* (DR max 35.10%) at 50.2 s, *Balsamic* (DR max 27.78%) at 59.6 s and *Bitterness* (DR max 25%) at 26.5s. However, the *Balsamic* attribute was the one that printed the longest time interval of the maximum dominance rate (T 90% max) with a duration of 16.9 s (Table 8).

In the olfactory evaluation of wines from the Cima Corgo sub-region, according to the interpretation of the TDS curves, the attributes: *Ripe Fruit*, *Floral*, *Balsamic*, *Spices*, and *Empyreumatic*, overlapped the line of significance level and were ordered according to the perception of the tasters. The highest maximum dominance rate was obtained by the attribute *Ripe fruit* (DR max 48.86%) at 8.3 s of the evaluation, successively by *Floral* (DR max 38.95%) at 16.9 s, *Empyreumatic* (DR max 37.88%) at 53.2 s, *Spices* (DR max 37.75%) at 50.3 s, and *Balsamic* (DR max 32.32%) at 27.2 s. The *Floral* attribute was expressed with the longest time interval of the dominance rate (T 90% max) with a duration of 4.8 s. Regarding the taste/flavor evaluation, the following attributes: *Astringency*, *Acidity*, *Fruity*, *Spices*, *Floral*, and *Balsamic*, overlapped the significance level line and were ordered according to the perception of the tasters. The highest maximum dominance rate was recorded by the attribute *Astringency* (DR max 41.67%) at 14.5 s of the evaluation, followed by *Fruity* attributes (DR max 37.88%) at 33.2s, *Spices* (DR max 37.37%) at 42 s, *Balsamic* (DR max 29.42%) at 54.7 s, *Acidity* (DR max 27.78%) at 13.5 s, and *Floral* (DR max 25%) at 48.5 s. The *Acidity* attribute was the one that printed the longest time interval of the maximum dominance rate (T 90% max) with a duration of 6.8s (Table 8).

In the olfactory evaluation of wines from the Douro Superior sub-region, according to the interpretation of the TDS curves (Figure 3), the attributes: *Flora*l, *Ripe Fruit*, *Balsamic*, *Spices*, and *Empyreumatic*, overlapped the line of significance level and were ordered according to the perception of the tasters. The highest maximum dominance rate was obtained by the attribute *Ripe fruit* (DR max 47.22%) at 8.5 s of the evaluation, successively by *Floral* (DR max 40.15%) at 21 s, *Balsamic* (DR max 34.85%) at 25.6 s, *Spices* (DR max 33.33%) at 31.5 s, and *Empyreumatic* (DR max 30.56%) at 36.5 s. The same was expressed with the longest time interval of the dominance rate (T 90% max) with a duration of 16.5 s. Regarding taste/flavor evaluation, the attributes of *Astringency*, *Fruity*, *Heat*, *Acidity*, *Floral*, *Spices*, and *Balsamic*, overlapped the significance level line and were ordered according to the tasters’ perception. The highest maximum dominance rate was recorded by the *Fruity* attribute (DR max 40.91%) at 31 s of the evaluation, followed by the attributes *Spices* (DR max 38.89%) at 47.5 s, *Astringency* (DR max 36.11%) at 7.5 s, *Acidity* (DR max 36.11%) at 20.5 s *Floral* (DR max 35.10%) at 4.2 s, *Balsamic* (DR max 34.97%) at 54.3 s, and *Heat* (DR max 29.34%) at 10.3 s. The *Astringency* attribute was the one that recorded the longest time interval of the maximum dominance rate (T 90% max) with a duration of 7.9 s (Table 8).

### 3.4. MANOVA Applied to TDS Data

To evaluate whether the sub-region had a statistically significant effect on the quantitative parameters (DR max, T max, and T 90% max), and concerning the olfactory characteristics, a MANOVA was performed for each of the attributes. The results obtained (Table 9) revealed statistically significant differences for the attributes *Fresh fruit* (λ_Wilks_ = 0.389, F_(6, 26)_ = 2.618, *p* = 0.04) and *Ripe fruit* (λ_Wilks_ = 0.344, F_(6, 26)_ = 3.054, *p* = 0.021). Regarding the *Fresh fruit* attribute, ANOVA revealed statistically significant differences for the DR max (F_(2, 15)_ = 9.064, *p* = 0.02) and T max (F_(2, 15)_ = 5.613, *p* = 0.015) parameters. Tukey’s post-hoc tests indicated significant differences for the DR max parameter between the Baixo Corgo and Cima Corgo sub-regions (*p* = 0.011) and Baixo Corgo and Douro Superior (*p* = 0.002). As for the T max parameter, the significant differences are between the Baixo Corgo and Douro Superior sub-regions (*p* = 0.017). Regarding the *Mature Fruit* attribute, ANOVA indicated statistically significant differences only for the T max parameter (F_(2, 15)_ =9.219, *p* = 0.002). Tukey’s post-hoc tests indicated that these differences occur between the sub-regions Baixo Corgo and Cima Corgo (*p* = 0.004) and Baixo Corgo and Douro Superior (*p* = 0.008).

Concerning the gustatory analysis (taste/flavor), a MANOVA was also performed for each of the attributes. The results obtained (Table 10) indicate significant statistical differences for the *Floral* retro-nasal attribute (λ_Wilks_ = 0.363, F_(6, 26)_ = 2.861, *p* = 0.028). The univariate variance analysis revealed statistically significant differences for the DR max parameter (F_(2, 15)_ = 4.565, *p* = 0.028). The Tukey post-hoc test indicated significant differences between the Baixo Corgo and Douro Superior sub-regions (*p* = 0.025).

## 4. Discussion

### 4.1. QDA^®^ of the Wines Sensory Profile

In the olfactory profile, the aromatic attributes, *Floral*, *Fruity*, *Balsamic*, *Spice*s, and *Empyreumatic* obtained a higher citation frequency rate both for PP1 and PP2 (Figure 2) in the evaluation of the wines of the three sub-regions. Except for *Empyreumatic*, which may be due to aromas related to the stage of wines in wooden containers, as well as furfurylthiol, a compound associated with the aroma of coffee [35], all other attributes have been already described by Böhm [22] and Almeida [23] in wines from Touriga Franca, Touriga Nacional, and Tinta Roriz grape varieties.

The SEM model (Figure 2), a statistical technique that proved to be valid and robust in the treatment of sensory data [20,36], was the technique of choice for our work. The model showed that, in the 2nd order factor analysis (Figure 2), the attributes *Aromatic Intensity* and *Aromatic Persistence* also contribute, in a representative way, to the sensory profile character of the wines of the three sub-regions, and converge between the PP1 and PP2 panels. Moreover, regarding the olfactory examination, for PP2, the *Type of Fruit* perception presented itself as *Fresh*, with a strong tendency towards *Ripe* only in the sub-region Baixo Corgo. In the other sub-regions, the attribute was registered as *Ripe* for both panels. Additionally, the descriptors mentioned could be placed on the bottle label, helping the consumers in the choice of the wine when purchasing.

Regarding the taste/flavor profile, it should be reported that, in the SEM 2nd order factor analysis, the taste/flavor attributes were the ones that presented the highest weight in all models.

The use of Factor Analysis (FA) allowed for verifying the homogeneity of the two panels. All tasters present high factor weights, which indicates a similar behavior. For the panel of trained tasters (PP1), the factor explains 89.716% of the total variance, while 92.163% is the percentage for specialists (PP2). In addition, the communality value for each of the tasters is high, indicating that the component is suitable for describing the latent factorial structure among the tasters. In other words, both panels were able to characterize the wines with a high level of objectivity.

### 4.2. TDS Curves and Wines Subregions Discrimination

The TDS technique consists of identifying and rating sensations perceived as dominant repeatedly until the perception end. It is important to note that the discussion of the results obtained is based on the results of the attributes that emerged above the significance level, both in the olfactory and taste/flavor evaluation. Pineau et al. [33] and Meillon et al. [34] studied the simultaneous evolution of several attributes over time and integrated the different perceptions into the temporal dominance of sensations (TDS) method, proving that, indeed, this method can be used to describe the temporality of wine sensations, and is an adequate methodology to identify wine quality descriptors.

In the olfactory evaluation, it was noticed that equally, for the three sub-regions of the DDR, the *Floral*, *Fruity*, and *Balsamic* attributes were expressed in the first part of the evaluations (Figure 3). As it was mentioned before, these attributes are described by Rogerson et al. [5], Böhm [22], and Almeida [23] as characteristics in the wines made from Touriga Franca, Touriga Nacional, and Tinta Roriz grape varieties. These grape varieties make part of the blend of the wines evaluated. *Spices* and *Empyreumatic* were expressed in the second part of the evaluations, according to the time of the maximum dominance rate (T max) of each attribute and can be related to aromas obtained after the wine stage in wooden containers [37,38]. *Floral* and the attributes related to the “type of fruit” stood out as the first two aromatic notes perceived by the tasters in the evaluations. *Floral* was revealed as an “attack note” in the wines of Baixo Corgo and *Ripe fruit* in the wines of Cima Corgo and Douro Superior sub-region.

Regarding the characterization of the “type of fruit”, in the Baixo Corgo sub-region, the attribute *Fresh fruit* with 40.54% of the evaluations was the one that recorded the highest maximum dominance rate. The Tukey Post-hoc tests indicated significant differences for this parameter between the sub-regions Baixo Corgo and Cima Corgo (*p* = 0.011), Baixo Corgo and Douro Superior (*p* = 0.002). However, in Cima Corgo and Douro Superior, it was the *Ripe Fruit* attribute that obtained the highest DR max, respectively, with 48.86% and 47.22% of the evaluations.

The *Spices* attribute obtained the longest time interval of the maximum dominance rate (T 90% max 20.15 s) in the evaluation of Baixo Corgo, while *Ripe Fruit* with 4.8 s in Cima Corgo and *Empyreumatic* with 16.5 s in the Douro Superior.

Regarding the taste/flavor evaluation, parity was shown between the sub-regions regarding the attributes *Astringency* and *Acidity*, being the first two to be identified and recorded in the first part of the tasting. Being that astringency is a highly relevant and dynamic attribute of wine quality, the TDS method is a sensory tool that can be applied to evaluate astringency over time [39]. Nevertheless, our results are divergent from what was obtained by the authors Etaio et al. [40] in their study to sensorily describe the red wines of the Rioja Alavesa region (the Spain Douro Region), where they report the same attributes as dominant, but, in the second moment of the evaluations.

Baixo Corgo wines recorded the highest maximum dominance rate (54.29%) for the attribute *Astringency*, followed by Cima Corgo (24.67%) and Douro Superior (36.11%). According to Meillon et al. [41], the decrease in alcohol concentration increases the domain of astringency. However, for Chacón-Vozmediano and co-authors [42], the quality of polyphenols, which confer the characteristic of astringency of wines, is associated with the degree of maturation and water feeding of grapevines. Excess water results in coarse polyphenols. That said, we highlight the correlation between the results indicated, the rainfall index, and the incidence of solar radiation in each sub-region (Table 1, in the introduction). Baixo Corgo also presented the highest DR max (50%) in the *Acidity* attribute. However, the attribute *Heat* (DR max 29.34%), related to alcohol perception, was pointed only in Douro Superior, which follows Jones [43] and Jones et al. [44]. The mentioned author states that the climate highly influences the characteristics of wines; wines from regions with a fresh climate present with pungent acidity, while alcohol is shown from high to very high for wines from hot and very hot regions. Vierra [45] also describes examples of the climatic relationship between the rise of alcohol, the decrease in acids, and the rise of pH in wines in his research.

The attributes, *Fruity*, *Spices*, and *Balsamic*, were recorded in the second part of the evaluations, contrary to the results obtained by the authors Medel-Marabolí et al. [15], in wines from Carmenère, Malbec, and Sangiovese grape varieties, where the *Fruity aroma* was recorded for the first time around initial 10 s of the evaluation. *Fruity* presented higher evidence (DR max) in Douro Superior with 40.91% of the evaluations.

The attribute *Floral*, with a 35.10% of maximum dominance rate, presented significant differences between the Baixo Corgo and Douro Superior sub-regions (*p* = 0.025).

*Balsamic* with 16.9 s in Baixo Corgo, *Acidity* with 6.8 s in Cima Corgo, and *Astringency* with 7.9 s in the Douro Superior, were the attributes that recorded the longest time interval of the maximum dominance rate (T 90% max).

## 5. Conclusions

The use of quantitative descriptive analysis and temporal dominance of sensations methods proved to be efficient in the wine’s sensory profile characterization. The QDA^®^ method, as described by other authors, was completed and detailed regarding the classification of attributes; thus, it is understood as an assertive choice for the same selection of attributes used in the TDS. Regarding the time required for the application of each of the methods, from training to the duration of each session, the TDS method proved to be much more efficient. TDS is a fast method that is easy to apply and has excellent results in the evaluation of the olfactory and taste/flavor profile of wines; however, it presents some limitations: (i) it restricts the evaluation of the wine’s visual characteristics and (ii) it is only focused on dominant sensations; thus, it is not possible to obtain information on all product attributes.

The wines of the three sub-regions have profiles with characteristics very similar in, olfactory, and taste/flavor aspects, pointing out a huge relation between the characteristics of the three sub-regions, Baixo Corgo, Cima Corgo, and Douro Superior, and the grape varieties Touriga Franca, Touriga Nacional, and Tinta Roriz. We can say that, globally, the olfactory profile of wines is characterized by *Fruity*, *Floral*, and *Balsamic* aromatic notes, while the taste/flavor profile stands out, highlighting *Astringency* and *Acidity* and again *Fruity* as the main in-mouth aroma. The descriptors mentioned could be a guide for consumers. When placed on the bottle label, it can help the consumers in their choice of wine when purchasing.

Regarding the behavior of the panelists, by the QDA^®^ method, it was noticed that, when compared to those trained, the specialists (experts) expressed more detailed results in the classification of qualitative attributes, such as *Type of Fruit*. Factor Analysis (FA) indicated homogeneity of the panels. For trained tasters, the factor explains 89.716% and, for the specialists, 92.163% of the total variance of data. The value of individual communality is high, revealing that the component is adequate to describe the latent factorial structure among the tasters. For all the sub-regions, the structural equation modeling (SEM) evidenced good adequacy.

Concerning the TDS, the expert tasters were at ease in carrying out the evaluations, both concerning the suggested evaluation protocol and the interface of the data acquisition software, which indicates that the methodology used in the training sessions was efficient. The use of multivariate analysis of variance followed by univariate analysis of variance revealed statistically significant differences, this being more relevant for the DR max parameter and higher maximum dominance rate.

Concerning a future perspective, the present work paves the way for new approaches that can contribute to the improvement and complement the study of the sensory profile of commercial wines through the methodologies used. As an example, we propose the highest number of samples aiming at increasing the relevance of results and integrating methods in the search for a rapid, detailed, less expensive, and at the same time, assertive evaluation. With a larger set of samples, it would be possible to obtain characteristic TDS curves for each Douro sub-region, providing a fingerprint for the wine that could be used for authentication and traceability purposes.

## Figures and Tables

**Table 1:** Climatic characteristics of the DDR (Demarcated Douro Region) sub-regions. Adapted from Magalhães [4].

Sub-Região	Altitude (m) ^1^ Lowest Quota	$\overset{\mathrm{IX}}{\underset{\mathrm{IV}}{\sum }}ta$ (Day) ^2^	R (mm) ^3^	Climate Classification
Baixo-Corgo	100	1.776	949	Humid
Cima-Corgo	130	1.926	672	Dry sub-humid
Douro Superior	150	2.241	407	semi-arid

^1^ Altitude in meters; ^2^ Incidence of annual solar radiation in hours; ^3^ Average annual rainfall in millimeters.

**Table 2:** List of wines used in the application of the QDA^®^ and TDS methodologies.

Sub-Region	SCTS	SCDP	Production Year	Grapes	Aged in Oak	ABV (% Vol.)	Total Acidity (g/L)	pH	Residual Sugar (g/L)
**Baixo Corgo**	249	BC01	2015	TN, TR, TC, TF	NI	14	5.3	3.56	NI
	124	BC02	2015	TN, TF, TR	Fob; 12 months	14	5.4	3.65	0.6
	704	BC03	2015	TF, TR, TN	Fob, Aob; 06 months	14	4.8	3.67	0.7
	680	BC04	2015	TF, TR, TA, AB, TFe, TN	Fob, 18 months	14	5.8	3.56	0.7
	106	BC05	2016	TN, TF, S, TC, TR	partial; Fob, Pob; 12 months	13.5	5.3	3.75	0.6
	684	BC06	2016	TN, TR, TF, OV	NI	14	NI	NI	NI
**Cima Corgo**	251	CC01	2015	TF, TN, TR	Fob; 12 months	14	5	3.7	2
	526	CC02	2015	TF, TN, TR, TC	Fob; 12 months	14	4.8	3.7	1.5
	713	CC03	2016	TN, TF, TR	Fob; 09 months	15	5.34	3.73	0.6
	860	CC04	2017	TF, TN, TR	partial (65%); Fob, Aob; 14 months	14	5.2	3.62	0.6
	506	CC05	2017	TN, TF, TR, TB	partial (15%); Fob; 06 months	14	4.9	3.73	1.8
	735	CC06	2017	TF, TN, TR, TC, TFra	Concrete tanks; Fob; 09 months	13.5	5.5	3.68	0.6
**Douro Superior**	497	DS01	2015	TN, TF, TR	partial (50%); Fob; 12 months	14	5	3.41	0.6
	951	DS02	2016	TN, TF, TR, TB, TC	partial; Fob; 10 months	14	5.4	3.75	NI
	895	DS03	2016	TN, TF, TR, TB	Fob; 10 months	14	5.2	3.65	0.6
	593	DS04	2016	TR, TF, TN, OV	Fob; 12 months	13.5	7	3.73	0.6
	682	DS05	2016	TN, TF, TR	partial; Aob; 06 months	14	5.3	3.7	0.7
	849	DS06	2017	TN, TF, TR, TA, TB	Fob; 12 months	14	4.1	3.66	2.2

SCTS—Sample code in test sessions; SCDP—Sample code in data processing; BC—Baixo Corgo; CC—Cima Corgo; DS—Douro Superior; ABV (% vol.)—Alcoholic strength by volume; pH—Hydrogen potential; Total Acidity—expressed in g/L of tartaric acid; Residual sugar—sugar content, expressed in g/L of glucose + fructose; TF—Touriga Franca; TN—Touriga Nacional; TFe—Touriga Fêmea; TR—Tinta Roriz; TC—Tinto Cão; TA—Tinta Amarela; TB—Tinta Barroca; TFra—Tinta Francisca; AB—Alicante Bouschet; S—Sousão; OV—Old Vines; ob—Oak barrels; Pob—Portuguese oak barrel; Fob—French oak barrel; Aob –American oak barrel; partial—when the wine batch has not been fully staged in wooden containers; NI—no information.

**Table 3:** List of attributes designated for application of the TDS methodology.

Olfactory Analysis (OA)	Taste/Flavor Analysis (TA)
Balsamic	Acidity
Empireumatic	Astringency
Spices	Bitterness
Floral	Balsamic
Fresh fruit	Heat
Ripe fruit	Spices
Fruit in jam	Floral
Dried fruit	Fruity

**Table 4:** Diagram of the content explained in the training sessions for the application of the QDA^®^ and TDS methods.

Training Session	QDA^®^	TDS
Session 01	Presentation of the research; Sensory tasting sheet to adapt to attributes and use of the tasting sheet	Introduction to the TDS method and its application; Presentation of data acquisition software; Familiarization test with the user interface of the data acquisition software, according to the print of the screens Training of the TDS Evaluation Protocol (OA, TA) (Table 5) with two samples of random red wines.
Session 02	Stimulation of olfactory perception through containers containing spices, fresh and dried fruits, and essences; Second sensory test to adapt to attributes and use of the tasting sheet	Stimulation of olfactory perception through containers with spices, fresh and dried fruits, and essences; Training of the TDS evaluation protocol with two samples of random red wines.
Session 03	Explanation of doubts; Stimulation of gustatory perception by tasting samples of adulterated wines reinforcing the understanding of attributes such as heat (sensation caused by alcohol), sweetness, acidity, bitterness, astringency, and mouth volume; Third sensory test to adapt to attributes and use of the tasting sheet	Clarification of doubts; Stimulation of gustatory perception by tasting samples of adulterated wines reinforcing the understanding of attributes such as heat (sensation caused by alcohol), sweetness, acidity, bitterness, astringency, and mouth volume; Third training of the TDS evaluation protocol with two samples of random red wines.

**Table 5:** TDS assessment protocol. Adapted from Pessina [31].

TDS Olfactory Assessment Protocol (OA)			TDS Taste/Flavor Assessment Protocol (TA)		
Stage	Time (s)	Instructions	Stage	Time (s)	Instructions
1	-	Remove the lid from the cup and hold it with your left hand.	1	-	Remove the lid from the cup and hold it with your left hand.
2	−5’	With the right hand start the evaluation by clicking the cursor in *Start* and shake the cup clockwise for 4’. If you have difficulty shaking the glass, use the table as a support base.	2	−5’	With the right hand start the evaluation by clicking with the cursor in *Start*; bring the wine to the mouth and make it evenly distributed, then discard. Do not evaluate this first contact with the wine.
3	0’	Smell the glass continuously for 8’ and at the same time click on one of the listed attributes that correspond to the most dominant at the moment. Click on a new attribute whenever you feel dominance change.	3	0’	Take the wine to the mouth and keep it for 4’; have it distributed evenly and at the same time click on one of the listed attributes that match the most dominant at the moment. Click on a new attribute whenever you feel dominance change. Wine can be swallowed or discarded.
4	from 9’ to 11’	Distance the cup from the nose; inhale and exhale for 2’; then re-smell the cup to continue the assignment of dominance by 7’.	4	from 5’ to 14’	Continue the evaluation and attribution of the dominant sensations by 9′.
5	from 18’ to 22’	Shake the glass clockwise for 4’. Then re-smell the glass to continue the assignment of dominance by 7’.	5	from 15’ to 19’	Repeat Step 3.
6	from 29’ to 31’	Repeat Step 4.	6	from 20’ to 29’	Repeat Step 4.
7	from 38’ to 42’	Shake the glass clockwise for 4’. Then re-smell the cup to continue the assignment of dominance by 6’.	7	from 30’ to 34’	Repeat Step 3.
8	from 48’ to 52’	Distance the cup from the nose; inhale and exhale for 2’; then re-smell the cup to continue the assignment of dominance by 8’.	8	from 35’ to 44’	Repeat Step 4.
9	60’	End of evaluation.	9	from 45’ to 49’	Repeat Step 3.
			10	from 50’ to 59’	Repeat Step 4.
			11	60’	End of evaluation.

**Table 6:** Component loadings, communalities, eigenvalue, and explained variance for PP1.

Tasters	Component Loadings	Communalities
Taster 1	0.936	0.875
Taster 2	0.940	0.884
Taster 3	0.910	0.829
Taster 4	0.915	0.837
Taster 5	0.975	0.950
Taster 6	0.960	0.921
Taster 7	0.950	0.902
Taster 8	0.953	0.909
Taster 9	0,943	0.889
Taster 10	0.938	0.880
Taster 11	0.931	0.866
Taster 12	0.954	0.911
Eigenvalue	10.766	
Explained variance	89.716%	

**Table 7:** Component loadings, communalities, eigenvalue and explained variance for PP2.

Tasters	Component Loadings	Communalities
Taster 1	0.929	0.862
Taster 2	0.974	0.949
Taster 3	0.944	0.890
Taster 4	0.969	0.940
Taster 5	0.972	0.945
Taster 6	0.972	0.944
Eigenvalue	5.530	
Explained variance	92.163%	

**Table 8:** Quantitative parameters of the TDS curves—Olfactory and taste/flavor analysis of the wines from the three sub-regions of the DDR. s—time in seconds.

Quantitative Parameters of TDS Curves									
**Olfactory Analysis**									
		Floral	Spices	Balsamic	Empyreumatic	Fresh fruit	Ripe fruit	Fruit in jam	Dried fruit
Baixo Corgo	*DR max*	46.09%	37.88%	32.58%	27.02%	40.45%	27.78%	2.78%	0%
	*T max*	8.7 s	44.8 s	28 s	55 s	14.3 s	58.5 s	7.5 s	0 s
	*T* 90*% max*	3.5 s	20.1 s	2.1 s	20 s	2.4 s	2.9 s	37.8 s	0 s
Cima Corgo	*DR max*	38.95%	37.75%	32.32%	37.88%	11.11%	48.86%	5.56%	0%
	*T max*	16.9 s	50.3 s	27.2 s	53.2 s	24.5 s	8.3 s	11.5 s	0 s
	*T* 90*% max*	4.8 s	2.2 s	1.8 s	4 s	1.4 s	2.8 s	4.2 s	0 s
Douro Superior	*DR max*	40.15%	33.33%	34.85%	30.56%	7.58%	47.22%	19.44%	0%
	*T max*	21 s	31.5 s	25.6 s	36.5 s	9 s	8.5 s	39.5 s	0 s
	*T* 90*% max*	9 s	3.5 s	3.6 s	16.5 s	1 s	5.1 s	3.3 s	0 s
**Taste/flavor analysis**									
		Astringency	Acidity	Heat	Bitterness	Spices	Fruity	Floral	Balsamic
Baixo Corgo	*DR max*	54.29%	50%	21.46%	25%	35.10%	38.13%	11.11%	27.78%
	*T max*	10.6 s	21.5 s	31 s	26.5 s	50.2 s	49 s	34.5 s	59.6 s
	*T* 90*% max*	3.8 s	4 s	3.6 s	3.8 s	2.8 s	4.5 s	7.4 s	16.9 s
Cima Corgo	*DR max*	41.67%	27.78%	16.67%	19.44%	37.37%	37.88%	25.00%	29.42%
	*T max*	14.5 s	13.5 s	45.5 s	56.5 s	42 s	33.2 s	48.5 s	54.7 s
	*T* 90*% max*	4.4 s	6.8 s	5.2 s	4 s	2 s	4.1 s	3.8 s	2.4 s
Douro Superior	*DR max*	36.11%	36.11%	29.34%	20.40%	38.89%	40.91%	35.10%	34.97%
	*T max*	7.5s	20.5 s	10.3 s	55.9s	47.5 s	31 s	41.2 s	54.3 s
	*T* 90*% max*	7.9 s	2.6 s	1.4 s	4.7 s	3 s	3.6 s	2.5 s	1.8 s

**Table 9:** Mean (M), standard deviation (SD), and multivariate significance of the TDS parameters—Olfactory analysis.

Attributes		Baixo Corgo	Cima Corgo	Douro Superior	*p*
		M ± SD	M ± SD	M ± SD	
Floral	*DR max*	0.647 ± 0.09	0.72 ± 0.158	0.667 ± 0.183	
	*T max*	20.500 ± 20.726	26.750 ± 13.842	21.667 ± 9.704	0.688
	*T*90*% max*	3.583 ± 3.932	2.733 ± 3.270	11.350 ± 14.967	
Spices	*DR max*	0.594 ± 0.176	0.611 ± 0.09	0.500 ± 0.182	
	*T max*	30.133 ± 14.261	39.850 ± 18.228	28.000 ± 13.882	0.267
	*T90% max*	6.817 ± 9.668	12.483 ± 17.467	16.400 ± 20.025	
Balsamic	*DR max*	0.528 ± 0.164	0.576 ± 0.146	0.492 ±0.107	
	*T max*	31.017 ± 19.704	30.500 ± 15.611	40.583 ± 15.700	0.660
	*T*90*% max*	13.517 ± 18.092	5.250 ± 7.969	7.800 ± 12.495	
Empyreumatic	*DR max*	0.417 ± 0.139	0.558 ± 0.119	0.576 ± 0.085	
	*T max*	39.017 ± 15.592	47.950 ± 12.294	44.767 ± 11.400	0.323
	*T*90*% max*	14.417 ± 12.027	4.200 ± 5.551	3.950 ± 2.421	
Fresh Fruit	*DR max*	0.528 ± 0.125	0.167 ± 0.258	0.083 ± 0.139	
	*T max*	18.167 ± 9.048	6.000 ± 10.164	3.000 ± 4.658	0.040
	*T*90*% max*	10.800 ± 17.478	1.067 ± 1.728	8.333 ± 15.726	
Ripe Fruit	*DR max*	0.361 ± 0.125	0.631 ± 0.276	0.545 ± 0.197	
	*T max*	46.533 ± 18.478	12.083 ± 7.826	15.383 ± 17.401	0.021
	*T*90*% max*	15.600 ± 18.746	20.100 ± 20.718	21.467 ± 23.627	
Fruit in jam	*DR max*	0.056 ± 0.086	0.083 ± 0.139	0.250 ± 0.139	
	*T max*	4.333 ± 7.560	11.850 ± 23.840	27.083 ± 15.272	0.147
	*T90% max*	7.600 ± 15.120	1.517 ± 2.360	7.033 ± 3.690	

**Table 10:** Mean (M), standard deviation (SD), and multivariate significance of the TDS parameters—Taste/flavor analysis.

Attributes		Baixo Corgo	Cima Corgo	Douro Superior	*p*
		M ± SD	M ± SD	M ± SD	
Astringency	*DR max*	0.768 ± 0.16	0.667 ± 0.182	0.661 ± 0.177	
	*T max*	14.050 ± 4.415	12.000 ± 4.183	16.833 ± 10.073	0.700
	*T90% max*	2.717 ± 1.392	3.467 ± 2.062	5.217 ± 5.206	
Acidity	*DR max*	0.703 ± 0.164	0.513 ± 0.120	0.583 ±0.175	
	*T max*	21.050 ± 5.112	14.500 ± 4.889	19.333 ± 4.875	0.341
	*T*90*% max*	2.200 ± 1.403	5.233 ± 7.411	4.583 ± 2.126	
Heat	*DR max*	0.371 ± 0.169	0.328 ± 0.175	0.447 ± 0.252	
	*T max*	32.550 ± 18.883	24.350 ± 21.205	14.950 ± 5.436	0.435
	*T*90*% max*	7.750 ± 12.263	12.583 ± 15.073	8.500 ± 15.590	
Bitterness	*DR max*	0.361 ± 0.222	0.250 ± 0.204	0.278 ± 0.172	
	*T max*	27.933 ± 19.076	36.367 ± 28.870	40.450 ± 24.090	0.225
	*T*90*% max*	7.867 ± 5.608	2.983 ± 2.369	9.533 ± 8.267	
Spices	*DR max*	0.528 ± 0.164	0.482 ± 0.138	0.495 ± 0.236	
	*T max*	42.517 ± 13.471	45.982 ± 10.033	48.800 ± 6.859	0.790
	*T*90*% max*	10.817 ± 10.653	8.167 ± 8.338	5.383 ± 7.129	
Fruity	*DR max*	0.649 ± 0.148	0.576 ± 0.100	0.667 ± 0.105	
	*T max*	28.033 ± 19.063	24.583 ± 10.052	26.517 ± 19.228	0.458
	*T*90*% max*	2.633 ± 1.645	4.767 ± 3.168	5.150 ± 3.210	
Floral	*DR max*	0.278 ± 0.09	0.444 ± 0.202	0.528 ±0.125	
	*T max*	40.350 ± 13.525	39.017 ± 16.110	46.683 ± 9.159	0.028
	*T*90*% max*	3.517 ± 1.990	10.817 ± 14.352	4.133 ±1.893	
Balsamic	*DR max*	0.409 ± 0.193	0.406 ± 0.118	0.566 ± 0.129	
	*T max*	53.300 ± 7.767	46.817 ± 17.888	48.400 ± 12.623	0.538
	*T*90*% max*	12.750 ± 18.707	16.100 ± 15.626	5.383 ± 6.097	

## Data Availability

Data is contained within the article or Supplementary Material.

## Supplementary Materials

The following supporting information can be downloaded at: https://www.mdpi.com/article/10.3390/foods11081168/s1, Table S1: List of descriptors used for the sensory profile of DOC Douro red wines.
