# Supplementary material for: Structural Equation Modeling (SEM) and Temporal Dominance of Sensations (TDS) in the Evaluation of DOC Douro Red Wine’s Sensory Profile
Source: Foods. 2022 Apr 18;11(8):1168. doi: 10.3390/foods11081168 (PMC9025624; doi:10.3390/foods11081168)
Supplement: Supplementary file 1 [file foods-11-01168-s001.zip › foods-1656561-supplementary.pdf]

Table S1. List of descriptors used for the sensory profile of DOC Douro red wines.

**Attributes**
**Visual Examination:**
Clarity
Color Intensity
Hue
**OlfactoryExamination:**
Aromatic Intensity
Floral
Fruity
Type of Fruit
Spices
Balsamic
Vegetable
Empireumatics
Minerals
Animals
Chemicals
Aromatic Persistence
**Taste Examination:**
Sweetness
Alcohol
Acidity
Astringency
Bitterness
Body
Balance
Taste Persistence
